# Combined Effect of Light and Nutrients on the Micromorphology of the White rot Fungus *Cerrena unicolor*

**DOI:** 10.3390/ijms21051678

**Published:** 2020-02-29

**Authors:** Anna Pawlik, Magdalena Jaszek, Dawid Stefaniuk, Urszula Świderska-Burek, Andrzej Mazur, Jerzy Wielbo, Piotr Koper, Kamil Żebracki, Grzegorz Janusz

**Affiliations:** 1 Department of Biochemistry and Biotechnology, Maria Curie-Skłodowska University, Akademicka 19 St., 20-033 Lublin, Poland; 2 Department of Botany, Mycology and Ecology, Maria Curie-Skłodowska University, Akademicka 19 St., 20-033 Lublin, Poland; 3 Department of Genetics and Microbiology, Maria Curie-Skłodowska University, Akademicka 19 St., 20-033 Lublin, Poland

**Keywords:** Light, micromorphology, *Cerrena unicolor*, arthrospores, sawdust, transcriptomes

## Abstract

Light influences developmental pathways in fungi. Recent transcriptomic and biochemical analyses have demonstrated that light influences the metabolism of a white-rot basidiomycete *Cerrena unicolor*. However, the expression profile of genes involved in the growth and development, or micromorphological observations of the mycelium in response to variable lighting and culturing media, have not performed. We aim to reveal the effect of light and nutrients on *C. unicolor* growth and a potential relationship between the culture medium and lighting conditions on fungus micromorphological structures. Confocal laser scanning microscopy and scanning electron microscopy were employed for morphological observations of *C. unicolor* mycelium cultivated in red, blue, green, and white light and darkness on mineral and sawdust media. A comprehensive analysis of *C. unicolor* differentially expressed genes (DEGs) was employed to find global changes in the expression profiles of genes putatively involved in light-dependent morphogenesis. Both light and nutrients influenced *C. unicolor* growth and development. Considerable differences in the micromorphology of the mycelia were found, which were partially reflected in the functional groups of DEGs observed in the fungus transcriptomes. A complex cross-interaction of nutritional and environmental signals on *C. unicolor* growth and morphology was suggested. The results are a promising starting point for further investigations of fungus photobiology.

## 1. Introduction

Sunlight is a very important signal for every living cell. It can be considered crucial for successful competition and survival in nature. Fungi use light as a source of information about the surrounding environment [1]. These microorganisms are equipped with several photosensory systems and can respond to different light intensities and colors [1,2,3]. For the perception of light, fungi employ various mechanisms comprising flavin-based blue-light sensors, retinal-based green-light sensors (such as rhodopsin), and linear tetrapyrrole-based red-light sensors, which suggests that they can detect specific wavelengths by discrete photoreceptory proteins [3].

Light affects the activity of numerous genes influencing metabolic and morphogenetic pathways related to many aspects of fungal life, i.e., control of developmental decisions, stress response, physiological adaptations, and the circadian clock [4]. Furthermore, light signaling is tightly linked with other pathways such as asexual sporulation, sexual development, primary metabolism, and the production of specific enzymes, demonstrating the complexity of light sensing and signal transduction in fungi [5,6]. Metabolite production is also strongly related to fungal morphology [7]. Spore formation and germination may be a result of the integration of many internal and external factors, e.g., light, air exposure, and mycelium nutritional stage. It may be presumed that, in the physiological sense, the transition from vegetative growth, which usually takes place in darkness, to the reproductive state (mostly occurring in light) requires drastic changes in fungal metabolism. Therefore, light signaling must be tightly connected with other signaling cascades, developmental pathways, and metabolic networks [1,4]. 

*Cerrena unicolor,* i.e., a wood-degrading basidiomycete of the Polyporaceae family, commonly known as a mossy maze polypore, is the causative agent of extensive white rot [8,9]. It bears the general features of fungi belonging to the genus *Trametes* [10]. Its fruiting body is characterized by a trimitic hyphal system producing single basidiospores [11]. This fungus is thought to be initially parasitic on living trees, but saprobic on dead wood. *C. unicolor* is found year-round, forming overlapping clusters on many deciduous hardwoods such as *Acer* spp., *Betula* spp., *Fraxinus excelsior*, *Fagus* spp., or *Quercus* spp. Moreover, strains of *C. unicolor* were also described as potential bioproducers of industrially-relevant enzymes [12,13,14,15,16] and other bioactive compounds with pharmacological and medical importance [17]. 

A recent RNAseq-based transcriptomic approach, followed by detailed biochemical analysis, demonstrated that light significantly influences *C. unicolor* metabolism, especially with respect to the production of enzymes engaged in wood degradation [6,18,19,20]. However, no comprehensive analysis of the expression profile of genes involved in fungal growth and development, nor any detailed micromorphological observations of fungus mycelium development or sporulation processes in response to light and culturing media, have been performed. Given the abilities of *C. unicolor* to degrade wood material and produce of biotechnologically significant compounds, a better understanding of fungal growth and morphogenesis may be extremely valuable. This could contribute to the characterization of the morphological changes induced in *C. unicolor* cells by variable conditions.

In this study, we aim to reveal the effect of light and nutrients on *C. unicolor* growth and the potential relationship between culture medium and lighting conditions on fungus micromorphological structures. A morphological observation of *C. unicolor* FCL139 cultivated in variable lighting conditions and growth media was performed. Differentially expressed genes and global changes in the expression profiles of *C. unicolor* genes putatively involved in morphogenesis and sporulation in response to light were also determined. A complex cross-interaction of nutritional and environmental signals affecting the growth and morphology of *C. unicolor* was postulated. The elucidation of changes occurring in *C. unicolor* cells contributing to a better understanding of the photobiology and light-dependent micromorphology of the fungus may be applied in future to improve the efficiency of biotechnological processes involving this environmentally and commercially important fungus. 

## 2. Results

In this work, morphological changes in white rot basidiomycete *C. unicolor* FCL139 triggered by different nutritional and lighting conditions (red, blue, green, and white light and darkness) were detected and assessed at a microscopic scale. Micromorphological observations of the mycelial structures and growth characteristics of the fungus were carried out using five-day-old *C. unicolor* aerial mycelium, cultivated in LH mineral medium and two different ash sawdust media in controlled lighting conditions. 

**Figure 1:**
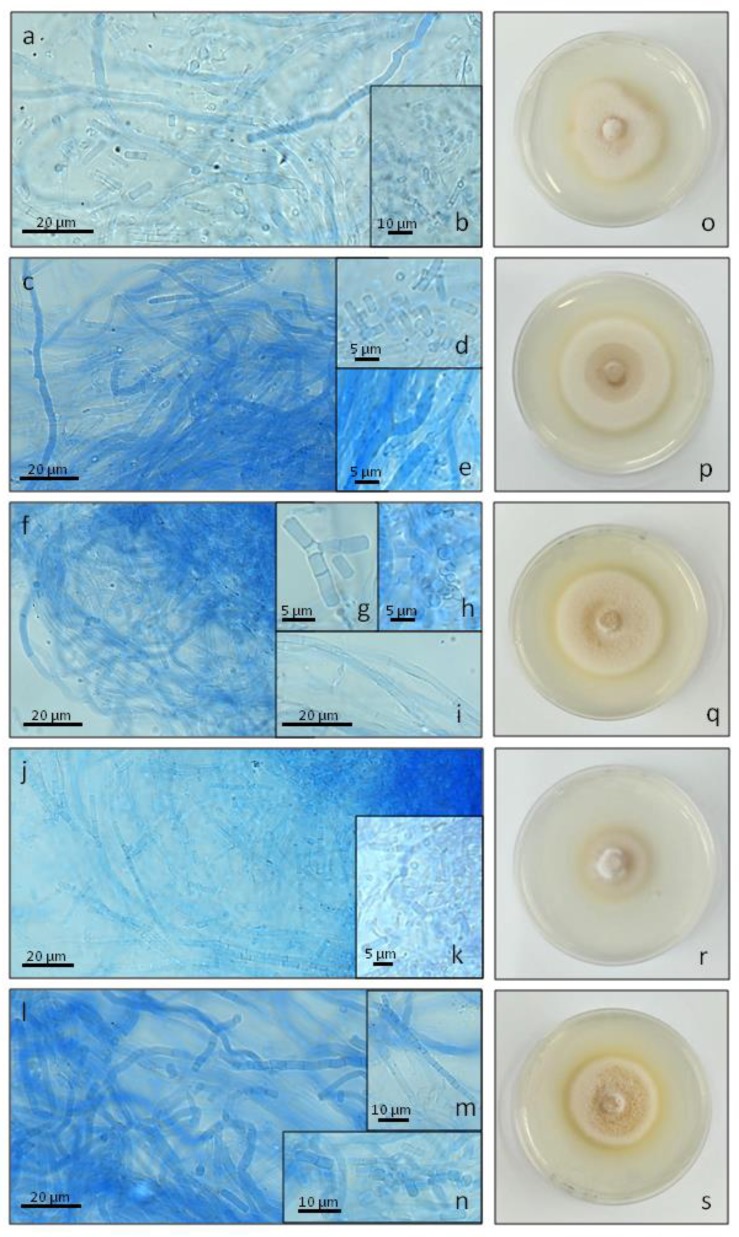
Confocal laser scanning micrographs of 5-day-old *Cerrena unicolor* cultures growing on 1.5% agar LH medium in white (**a**,**b**), dark (**c**–**e**), red (**f**–**i**), blue (**j**,**k**), and green (**l**–**n**) light conditions; Photographs of *C. unicolor* 5-day-old cultures growing on 1.5% agar LH medium in 90 mm Petri plates in white (**o**), dark (**p**), red (**q**), blue (**r**), and green (**s**) light conditions.

In general, both the light and culturing conditions influenced *C. unicolor* growth and development. In the Petri dish cultures, *C. unicolor* exhibited typical rapid radial growth with concentric zones, and formed a pure white mat, which was more compact when light (regardless of the wavelength) was applied, in comparison to darkness. Moreover, a slightly irregular growth zone was observed only in the white lighting conditions. The smallest growth zone, i.e., 43 mm, indicating the weakest fungal growth, was observed in the blue light conditions. The fastest growth was observed in darkness, as the mycelium reached a diameter of 63 mm within 5 days (Table 1 and Figure 1o–s).

**Figure 2:**
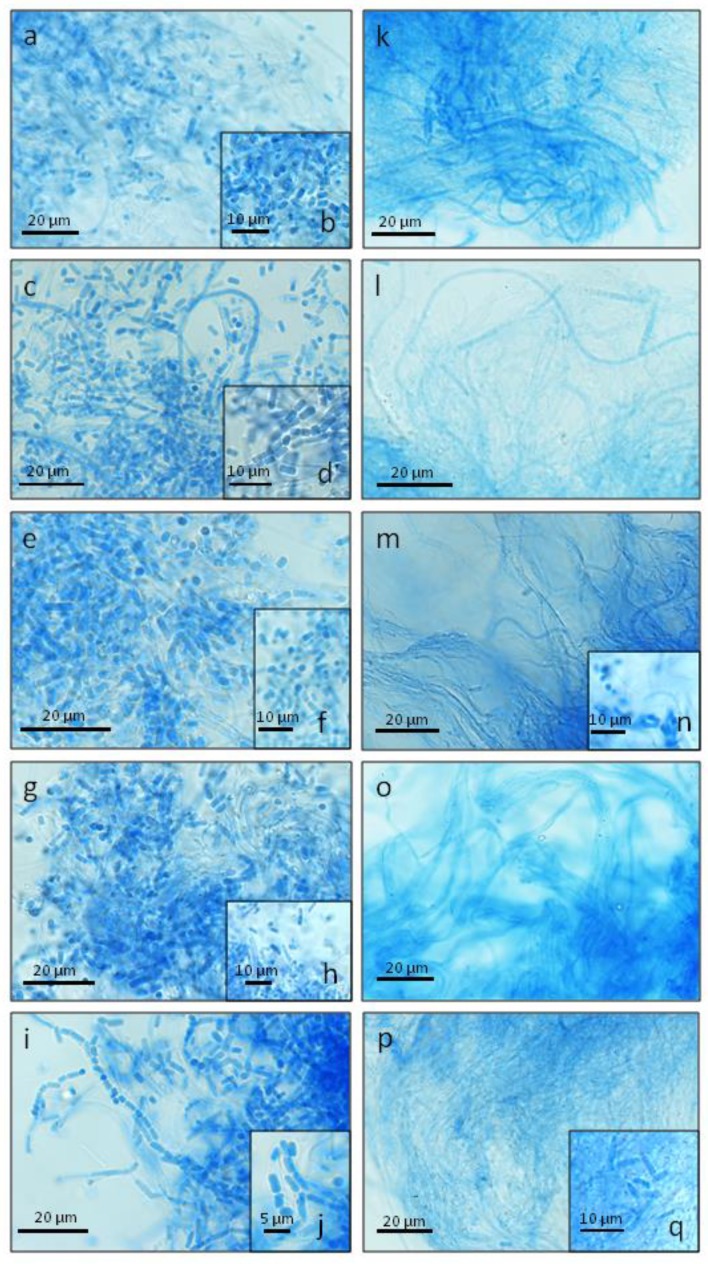
Confocal laser scanning micrographs of 5-day-old *Cerrena unicolor* cultures growing on 1.5% agar ash sawdust medium in white (**a**,**b**), dark (**c**,**d**), red (**e**,**f**), blue (**g**,**h**), and green (**i**,**j**) light conditions; Confocal laser scanning micrographs of 5-day-old *C. unicolor* cultures growing on ash sawdust medium in white (**k**), dark (**l**), red (**m**,**n**), blue (**o**), and green (**p**,**q**) light conditions.

*Cerrena unicolor* exhibited considerable differences in the morphological features of the mycelia, e.g., the width of hyphae or number and size of spores, in the different culturing conditions tested in this work (Figure 1 and Figure 2, Table 1). Lactophenol Cotton Blue staining performed for *C. unicolor* aerial mycelium, followed by confocal laser scanning microscope (CLSM) analysis, highlighted the presence of typical skeletal hyphae, hyphae with single septa, branching hyphae, and septated hyphae, producing arthrospores. The width of this structures was variable and apparently dependent on the lighting conditions and growth medium applied during cultivation (Table 1). In general, unbranched skeletal hyphae and hyphae with single septa predominated, and were the only types observed in all culturing variants, while the occurrence of the other hyphal types was variable and dependent on the culturing conditions. It is worth noting that rare branched hyphae were formed only in darkness, both on the mineral and sawdust media. No significant differences in the structure and size of the hyphal system were demonstrated (Table 1). Microscopic imaging revealed the presence of numerous thin-walled arthrospores. These can be described as cylindrical to subcylindrical or broadly ellipsoid in shape; their amount depended on the growth medium and light wavelength (Figure 1 and Figure 2). In all culturing variants, predominantly cylindrical arthrospores were produced. Broadly ellipsoid spores were rather rarely observed. In general, the spores had dimensions of 3.13–9.3 × 1.92–3.06 μm (on average), except for the cultures grown in white light, where they were the smallest (Table 1). Quantitatively, the most abundant sporulation was observed in the 1.5% agar ash sawdust medium and the blue/white light conditions. The smallest number of arthrospores was observed in the ash sawdust medium, where predominantly long and thin, mainly skeletal types of hyphae were produced (Figure 2).

**Figure 3:**
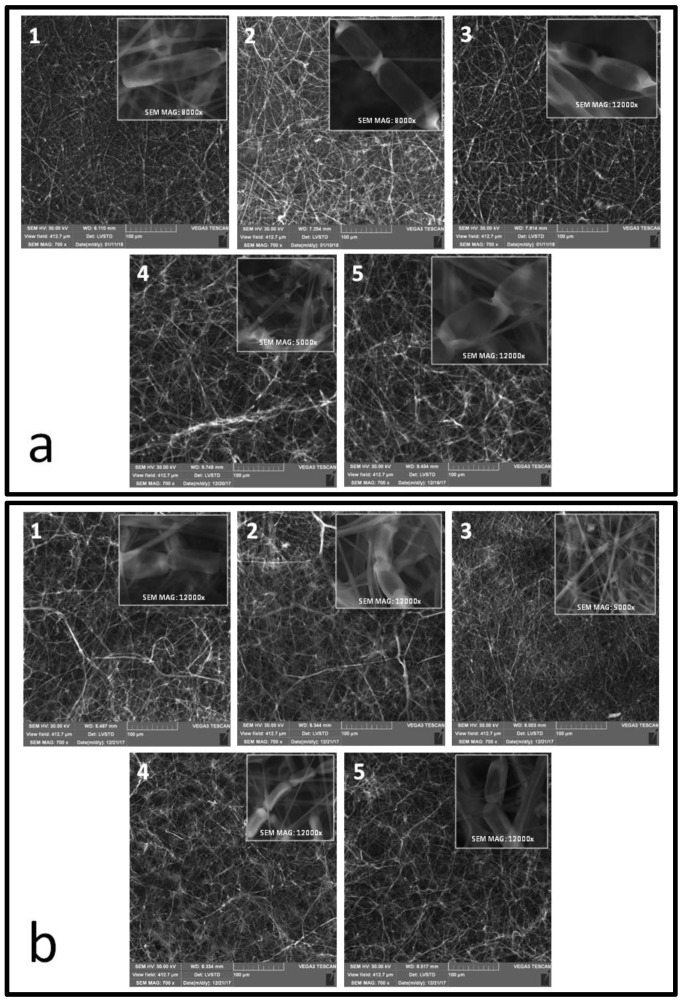
SEM micrographs of the surface of *Cerrena unicolor* mycelium growing on 1.5% agar LH (**a**) and ash sawdust (**b**) media observed in white (1), dark (2), red (3), blue (4), and green (5) light conditions.

In order to visualize the surface structure of the *C. unicolor* mycelium, scanning electron microscopy (SEM) was applied. The SEM micrographs confirmed our previous CLSM data and showed typical hyphal system including septated hyphae producing arthrospores (Figure 3). The distribution of the hyphae was rather regular with numerous intercrosses creating clumps. The differences in the mycelium morphological features between the lighting conditions were especially noticeable for *C. unicolor* cultured in darkness on the sawdust substrate, where the fungus formed wide branching hyphae (Figure 3b). In turn, cultivation in red light caused the formation of a regular network of hyphal structures as well as relatively long and thin unbranched hyphae. Due to the vacuum mode applied for the SEM analyses, no single arthrospores could be observed.

**Figure 4:**
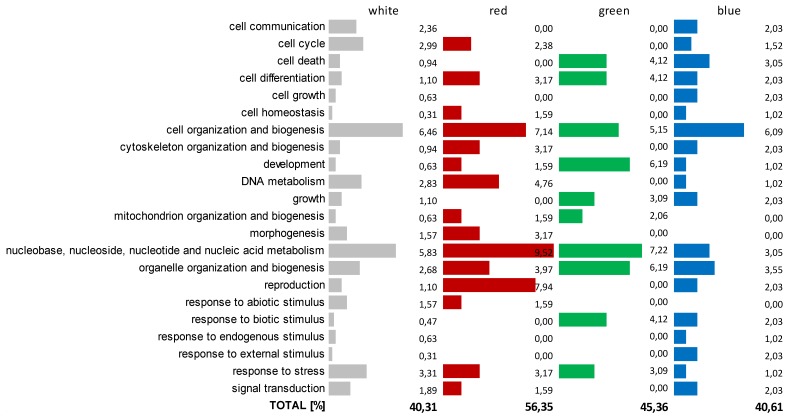
GO annotation of *Cerrena unicolor* 451 DEGs putatively engaged in fungal growth, development, and morphology. Color bars represent % of DEGs specific for fungus cultivation in different lighting conditions, which received GO annotations referring to biological processes related with *C. unicolor* growth and morphology. DEGs were grouped into nine functional categories: cellular process, developmental process, growth, homeostatic process, cellular component organization and biogenesis, primary metabolic process, metabolic process, reproduction, and response to stimulus (numerous DEGs were classified into more than one GO category). Only DEGs whose abundance among GO terms exceeded 1% for at least one lighting variant were presented.

Next, we tried to find some potential correlation between the results of the micromorphological changes in the *C. unicolor* hyphae and spores observed in this work during fungus growth in various lighting conditions and the differential gene expression confirmed previously [6] in the same cultivation conditions. Among the numerous differentially expressed genes (DEGs) observed in the transcriptome analysis of *C. unicolor* growing in white, red, blue, and green light in comparison to darkness, 451 DEGs were functionally classified with GO (Gene Ontology) terms and placed into GO Slim categories, comprising nine functional groups related to fungal growth and development (Figure 4). DEGs related to fungal growth and development constituted a significant part of the total number of differentially expressed genes assigned with GO terms, and varied substantially in the transcriptomes of *C. unicolor*, i.e., from 40.31% in white light treatment to 56.35% of DEGs in the red light variant. The most abundant functional groups into which the largest number of DEGs was classified were cellular processes and cellular component organization or biogenesis categories. Transcripts of DEGs related to the ‘cell organization and biogenesis’ category were the most widely represented in the transcriptomes of the fungus, regardless of the lighting variant, and ranged from 5.15% for the green light up to 7.14% for red light conditions (Figure 4). Among the GO classification categories, DEGs related to ‘nucleobase, nucleoside, nucleotide, and nucleic acid metabolism’ (9.52%) predominated in the transcriptome of *C. unicolor* grown in red light (Figure 4). Surprisingly, compared to the other variants, ‘reproduction’ and ‘development’ were the dominant categories of DEGs in the red (7.94%) and green light (6.19%) conditions, respectively.

## 3. Discussion

Fungal development and metabolite production comprise highly controlled processes which are frequently modified by environmental factors such as light, which may control morphological, physiological, and metabolic responses in fungi. Such regulation is a consequence of millions of years of fungal evolutionary adaptation to illumination by the sun. Developmental responses of multicellular organisms to external stimuli entail complex interactions between gene expression and mechanisms of physiological control [4,5,21].

In the present work, microscopic observations of morphological features of *C. unicolor* cultured in vitro in different lighting conditions and growth substrates were performed. To date, the microcharacteristics of the mycelium structure of this fungus have been reported only from dark-grown cultures. The *C. unicolor* fruiting body was characterized by a trimitic hyphal system with thin-walled generative hyphae, skeletal hyphae with thick walls, and branched binding hyphae [11]. The recorded widths of hyphae from different growth zones cultivated in vitro were 1.5–5 μm, 1.5–5 μm, and 1–2.5 μm for the advancing zone, submerged zone, and fiber (skeletal) hyphae from aerial mycelium, respectively [11,22,23]. It was also observed that *C. unicolor* produced in vivo single, ellipsoidal, hyaline, smooth, and thin-walled basidiospores of 4.5–5.5 × 2.5–3.5 µm in size [11] with in vitro induction of arthrospore production [22].

In the Petri dish cultures, *C. unicolor* formed a pure white mat with typical concentric zones (Figure 1), as described elsewhere [11,23]. Fungal growth proved to be more compact in the light than in the darkness conditions. Regardless of the growth conditions, the in vitro widths of hyphae of the five-day-old aerial *C. unicolor* mycelium correlate well with previous descriptions of this type of fungal cultures [11,23]. The overall morphological features also agreed with the fungus mycelium structure observed in SEM [24]. The more compact *C. unicolor* hyphal growth in the light than in the darkness conditions may be related to the expression of *wc* (white collar) genes encoding proteins of a blue light receptor family, also acting as transcription factors of other light-inducible genes [3,25]. Interestingly, transcripts coding for putative white collar proteins have already been identified in *C. unicolor* transcriptome during fungus growth in different lighting conditions [6]. It has been demonstrated that hyphal growth may require expression of *wc* genes [25], which may further suggest a similar regulatory pathway of *C. unicolor* growth. The regulation of hyphal branching by light has been described in *Neurospora crassa* [26], *Tuber borchii* [27], and *Trichoderma atroviride* [28]. This was found to result in fungal colonies that were more compact during growth under light. However, in this work, the presence of branched hyphae typical for light-grown mycelium was observed in the dark-grown *C. unicolor* colonies (Table 1, Figure 1 and Figure 3). The engagement of additional metabolic signals in such a phenomenon cannot be excluded, as in *Hypocrea atroviridis*, where cross-talk between blue light receptors, carbon metabolism, and response to oxidative stress has been described [29].

Microscopic imaging of the *C. unicolor* mycelium biomass revealed the presence of numerous thin-walled arthrospores (Figure 2 and Figure 3), which were generally formed as a result of segmentation and subsequent fragmentation of existing hyphae [30]. The amount of arthrospores proved to be dependent on the light wavelength and the growth medium (Figure 1 and Figure 2). This observation suggests the existence of additional metabolic signals for fungal morphogenesis. It has been demonstrated that morphogenesis in fungi is often induced by extracellular factors and executed by respective encoded fungal effectors [7,31]. Light-dependent asexual photoconidiation has already been reported for *N. crassa* [32], *Paecilomyces fumosoroseus* [33], *Aspergillus nidulans* [5], or *Trichoderma reesei* [34], and specific response mechanisms involving light-sensitive elements have been proposed. However, it should be considered that light and its wavelength can exert opposite effects in different fungi [25,35,36]. This is also not surprising if one considers arthrospore production in fungi as a result of metabolic stress [25,30]. Light, especially blue wavelengths, has been described as the most effective stress factor in fungal photomorphogenesis [25]. Some culture conditions used in the current experiments were potentially stressful, as evidenced previously by the activity of antioxidant enzymes (superoxide dismutase and catalase) determined in *C. unicolor* cells cultivated in the same lighting and nutritional conditions [20]. Nevertheless, the largest number of spores in *C. unicolor* cultures were formed in the 1.5% agar ash sawdust medium and blue/white light conditions (Figure 2), which may suggest a potential cross-talk between blue light receptors and perception of metabolic signals derived from the growth medium. In *N. crassa*, photoregulation of *cot-1* (kinase-encoding gene involved in hyphal growth) transcription was shown to be dependent on the carbon source employed in the culture medium [26]. The finding that deviations from optimal culture conditions, likewise changes in carbon sources [29], can further modulate the light effect on fungal growth [37] and metabolism [18] indicates that the responses to the availability and quality of the carbon source and light are closely interlinked [4]. 

Four hundred and fifty-one DEGs were classified with GO (Gene Onthology) terms into nine functional groups related to fungal growth and development, which constitutes a significant part of the total number of differentially expressed genes observed in the transcriptome analysis of *C. unicolor* cultivated in different lighting conditions (Figure 4). The largest number of DEGs was assigned to cellular processes and cellular component organization or biogenesis categories, which shows high importance of such pathways in fungal morphogenesis. However, no direct correlation between certain GO categories, morphological features, and lighting conditions could be indicated. Such a potential correlation can be found between the fungal life cycle and adaptation of its reproduction style, where stage- and light-specific gene expression occurs [5], or during the development of the fruiting body [38].

## 4. Materials and Methods 

### 4.1. Strain, Medium, and Cultivation Conditions

The *C. unicolor* (Bull. ex Fr.) Murr. strain was obtained from the Botany Institute II, Regensburg University, Regensburg, Germany, and deposited in the Fungal Culture Collection (FCL) of the Department of Biochemistry and Biotechnology, Maria Curie–Skłodowska University, Lublin, Poland as strain FCL139 [39]. The stock culture was maintained in 2% (*w/v*) malt extract agar (Difco, BD, NJ, USA) slants. As an inoculum, ca. 5 mm^2^ of the slant was punched out with a sterilized cutter. Then, the mycelium was transferred into a 50-mL liquid Lindeberg-Holm [40] (LH) medium in a 100-mL Erlenmayer flask. The seeds were cultivated in the dark at 28 °C. Next, ten-day-old mycelia were homogenized in a disperser homogenizer T18 basic ULTRA-TURRAX (IKA, Staufen, Germany), and 0.6 mL of the homogenate was used to inoculate the experimental cultures as described below. The mycelia were cultivated in three medium variants, i.e., (I) 1.5% agar LH medium, (II) 1 g of sterile ash sawdust (wood particles < 4 mm) soaked with 9 mL of distilled water, and (III) solidified 10 % ash sawdust medium in Petri dishes at 28 °C in incubators equipped with LED illumination cassettes (KT 115, Binder, Tuttlingen, Germany). Continuous light conditions (20 lux) were provided throughout the *C. unicolor* cultivation period. The following light variants were applied: white (color temperature 4000–4750 K), green (510–520 nm), blue (465–470 nm), and red (620–625 nm) light and darkness as a control. Microscopic observations of micromorphological structures were performed using five-day-old mycelium.

### 4.2. Visualization of the Micromorphology of Hyphae Using the Confocal Laser Scanning Microscope (CLSM)

To determine the micromorphological structures of *C. unicolor* fungal cells, separate pieces of aerial mycelia (2.5 mm in diameter) were cut from the fungal biomass and stained with Lactophenol Cotton Blue. Then, the mycelium fragments were placed in a drop of the dye on a basic glass slide, covered with a coverslip, and gently heated. An inverted microscope, the Axiovert 200M (Carl Zeiss Light Microscopy, Jena, Germany) equipped with an LSM5 Pascal head (magnification 200×), was used for visualization of the *C. unicolor* mycelium micromorphology.

### 4.3. Scanning Electron Microscope (SEM) Analysis of the Structure of Fungal Hyphae 

In order to visualize the surface structure of the *C. unicolor*, mycelium pieces of aerial mycelia (5 mm^2^) were gently separated from medium residues and placed on aluminum stubs covered with a double-sided tape. Uncoated samples were imaged in SEM (VEGA3 LM, Tescan, Brno, Czech Republic) in a low vacuum mode in a frozen state (Peltier’s cooled stage, pressure 15 Pa, temperature in the range from −35 °C to −45 °C).

### 4.4. Data Analysis

At least nine fragments (2.5 mm in diameter) of the *C. unicolor* aerial mycelium were used for all microscopic analyses performed in three biological replications per each experimental light variant.

### 4.5. Analysis of the Expression Profile of Genes Engaged in Fungal Growth and Development

In the search for the expression profile of genes putatively involved in fungal growth and development, we reanalyzed the data on the differential gene expression during *C. unicolor* cultivation in various lighting conditions (darkness, white, red, blue, and green light) obtained in the RNAseq experiment and described previously [6]. Individual differentially expressed genes were placed into GO Slim categories using Gene Ontology terms, which were ascribed to individual transcripts based on their automated annotations and predicted sequence similarities to known proteins of SwissProt and TrEMBL databases, as well as the presence of conserved domains (PFAM database). To reveal the expression profile of genes engaged in fungal growth and development which were differentially expressed in tested lighting condition, we focused on DEGs classified into one of the functional groups putatively engaged in cellular processes, developmental processes, growth, homeostatic processes, cellular component organization and biogenesis, primary and secondary metabolic processes, reproduction, and response to various biotic and abiotic stimuli. 

## 5. Conclusions

In summary, we assessed, for the first time at the microscopic level, the morphological changes triggered in *C. unicolor* by different lighting and nutritional conditions, which were also partially reflected in differential gene expression at the transcriptomic level. The results suggest a complex cross-interaction of nutritional and environmental factors on the growth and morphological features of *C. unicolor*. A potential relationship between the growth medium and light culture conditions on fungus micromorphological structures was demonstrated. Nevertheless, taking into consideration the complexity of the photomorphogenetic pathways, these data should be considered as a promising starting point for more detailed investigations. This will be a necessary step towards the elucidation of the *C. unicolor* photobiology and the application of such knowledge to effect efficient improvements of industrial and biomedical processes employing this fungus.

## Figures and Tables

**Table 1:** Overall characteristics of *Cerrena unicolor* morphology observed in white, dark, red, blue, and green light conditions in different growth media.

**1.5 % agar LH Synthetic Medium**	**Lighting Conditions**				
	**White**	**Darkness**	**Red**	**Blue**	**Green**
Mycelium dimensions (mm × mm)	56 × 55 ± 1.6	63 × 63 ± 1.2	60 × 61 ± 0.6	43 × 43 ± 1.7	58 × 60 ± 0.8
Hyphae type and size (width, μm)	Skeletal hyphae (1.62–2.35); hyphae with single septa (2.03–3.33); septated hyphae producing arthrospores (1.86–3.3)	Skeletal hyphae (1.7–2.6); hyphae with single septa (2.76–3.98), branched hyphae (2.7–3.68) (rare); septated hyphae producing arthrospores (1.7–2.92)	Skeletal hyphae (1.78–2.6); hyphae with single septa (1.95–3.9); septated hyphae producing arthrospores (2.03–3.49)	Skeletal hyphae (1.7–2.35); hyphae with single septa (2.27–3.57); septated hyphae producing arthrospores (1.62–3.00, dominance)	Skeletal hyphae (1.9–2.7); hyphae with single septa (3.0–3.9); septated hyphae producing arthrospores (2.43–3.25)
Spore shape	Cylindrical (dominance); broadly ellipsoid	Cylindrical (dominance); broadly ellipsoid	Cylindrical (dominance); broadly ellipsoid	Cylindrical (dominance); broadly ellipsoid	Cylindrical (dominance); broadly ellipsoid
Spore size [μm × μm]	3.0–7.0 × 1.7–3.0	3.0–11 × 2.0–3.5	3.5–10 × 2.0–3.5	3.5–13 × 1.5–3.5	2.5–8.5 × 2.0–3.5
**1.5 % Agar Ash Sawdust Medium**	**Lighting Conditions**				
	**White**	**Darkness**	**Red**	**Blue**	**Green**
Hyphae type and size (width, μm)	Skeletal hyphae (1.54–2.03); hyphae with single septa (1.94–2.63); septated hyphae producing arthrospores (1.54–2.51, rare)	Skeletal hyphae (1.7–2.51); hyphae with single septa (2.53–3.8); septated hyphae producing arthrospores (2.11–2.76, rare); branched hyphae (2.35–3.5, rare)	Skeletal hyphae (1.46–2.11); septated hyphae producing arthrospores (1.54–2.35, rare); hyphae with single septa (1.96–3.25, rare)	Skeletal hyphae (1.54–2.43); septated hyphae producing arthrospores (1.86–3.0, rare)	Skeletal hyphae (1.46–2.27, dominance); hyphae with single septa (1.95–3.65); septated hyphae producing arthrospores (1.82–2.61)
Spore shape	Cylindrical (dominance); broadly ellipsoid	Cylindrical (dominance); broadly ellipsoid	Cylindrical (dominance); broadly ellipsoid	Cylindrical (dominance); broadly ellipsoid	Cylindrical (dominance); broadly ellipsoid
Spore size (μm × μm)	2.5–6.0 × 2.0–3.0	2.0–14 × 2.0–2.5	4.0–13 × 2.0–3.0	3.5–10 × 2.0–3.0	3.0–7.5 × 2.0–3.0
**Ash Sawdust Medium**	**Lighting Conditions**				
	**White**	**Darkness**	**Red**	**Blue**	**Green**
Hyphae type and size (width, μm)	Skeletal hyphae (1.54–2.27); hyphae with single septa (1.7–2.63); septated hyphae producing arthrospores (1.54–3.0)	Skeletal hyphae (1.46–2.6); septated hyphae producing arthrospores (1.62–2.27); hyphae with single septa (1.91–2.7, rare)	Skeletal hyphae (1.48–2.27) (dominance); hyphae with single septa (1.7–2.63); septated hyphae producing arthrospores (1.66–2.68)	Skeletal hyphae (1.62–2.54, dominance); hyphae with single septa (1.7–3.3)	Skeletal hyphae (1.54–2.03, dominance); hyphae with single septa (1.7–3.12); septated hyphae producing arthrospores (1.62–2.6, rare)
Spore shape	Cylindrical (dominance); broadly ellipsoid	Cylindrical (dominance); broadly ellipsoid	Cylindrical (dominance); broadly ellipsoid	Cylindrical (dominance); broadly ellipsoid	Cylindrical (dominance); broadly ellipsoid
Spore size (μm × μm)	3.0–6.0 × 1.7–2.5	3.5–10 × 2.0–3.0	3.0–8.5 × 2.0–3.0	3.5–8.5 × 2.0–3.0	3.5–7.5 × 2.0–3.0

## Abbreviations

DEGs	Differentially Expressed Genes
GO	Gene Ontology
CLSM	Confocal Laser Scanning Microscope
SEM	Scanning Electron Microscope
